# Compound Heterozygous *MRPS14* Variants Associated With Leigh Syndrome

**DOI:** 10.1002/acn3.70065

**Published:** 2025-05-02

**Authors:** Maria Gabriela Otero, Christina Freeman, Ruchi Shah, Renkui Bai, Hong Cui, Marian Castro, Zachary Myers, Eric Choy, Derek Chan, Molly Easter, Sophia Y. Zhao, Madeline Babros, Ruchi Garg, Matthew Deardorff, Franklin Moser, Tyler Mark Pierson

**Affiliations:** ^1^ Board of Governors Regenerative Medicine Institute Cedars‐Sinai Medical Center Los Angeles California USA; ^2^ Biomedical Sciences Cedars‐Sinai Medical Center Los Angeles California USA; ^3^ Regenerative Medicine Institute Eye Program Cedars‐Sinai Medical Center Los Angeles California USA; ^4^ GeneDx Gaithersburg Maryland USA; ^5^ Pediatric Congenital Heart Program at Cedars‐Sinai Guerin Children's and Smidt Heart Institute Cedars‐Sinai Medical Center Los Angeles California USA; ^6^ Department of Pathology and Laboratory Medicine Children's Hospital Los Angeles California Los Angeles USA; ^7^ Division of Clinical Neuroradiology and Interventional Neuroradiology Cedars‐Sinai Medical Center Los Angeles California USA; ^8^ Division of Pediatric Neurology, Department of Pediatrics Cedars‐Sinai Guerin Children's, Cedars‐Sinai Medical Center Los Angeles California USA; ^9^ Department of Neurology Cedars‐Sinai Medical Center Los Angeles California USA

**Keywords:** Leigh Syndrome, mitochondrial ribosome, MRPS14, oxidative phosphorylation

## Abstract

MRPS14 (uS14m) is a nuclear‐encoded ribosomal protein important for mitochondria‐specific translation. To date, only a single individual with a recessive *MRPS14*‐related disorder (also known as COXPD38) has been reported. We report an additional subject possessing novel compound heterozygous *MRPS14* variants (p.Asp37Asn, p.Asn60Asp). The subject presented at 2 years with motor and language delays associated with elevated serum lactate/alanine levels. Brain MRI showed a constellation of signal abnormalities consistent with Leigh Syndrome, while MR spectroscopy had an increased lactate peak. Western blots of fibroblasts showed decreased MRPS14 and COX2 protein levels. These results support the pathogenicity of the MRPS14 variants identified here.

## 1 Introduction

Mitochondria manifest several unique organellar features beyond the generation of cellular energy via oxidative phosphorylation (OXPHOS) [1]. Mitochondria maintain an independent circular genome (mtDNA) that is replicated via a prokaryotic‐like process [2, 3]. Mitochondria also utilize a dedicated and unique transcriptional and translational apparatus for expressing mtDNA‐encoded proteins and RNAs, which includes 13 mt‐proteins, 22 mt‐tRNAs, and 2 mt‐rRNAs [2, 3]. Many mtDNA‐encoded proteins (mt‐proteins) are vital parts of the electron transport chain complexes that are required for OXPHOS; therefore, dysfunction of any mitochondrial‐specific translational protein may lead to significant clinical disease [2, 3, 4, 5, 6, 7, 8, 9].

Mitochondrial‐specific ribosomes (mt‐ribosomes) are composed of a large 39S subunit and a small 28S subunit [4, 5, 6]. The 39S subunit consists of a 16S‐mt‐rRNA and 50 different mitochondrial ribosomal large subunit proteins (MRPLs). The 28S subunit is composed of a 12S mt‐rRNA and 30 different mitochondrial ribosomal small subunit proteins (MRPSs) [7, 8]. *MRPL* and *MRPS* genes are nuclear‐encoded, with only 10 of these genes being linked with human disorders (*MRPL3*, *MRPL12*, *MRPL44* and *MRPS2*, *MRPS7*, *MRPS14*, *MRPS16*, *MRPS22*, *MRPS23*, *MRPS34*) [4]. These disorders often present in early childhood in a heterogeneous and multi‐systemic manner, that can include liver and renal dysfunction, myopathies, cardiomyopathies, brain malformations, along with neurological and neurodevelopmental disabilities [4]. Common neurological features include global developmental delay, seizures, ataxia, and progressive neurodegeneration [10].

Recently, a consanguineous child (Subject‐R108C) was reported with homozygous variants (c.322C>T; p.Arg108Cys) causing an *MRPS14*‐related disorder (also known as COXPD38: OMIM #618378) [4]. Subject‐R108C was found to have diffuse hypotonia, motor delays, dysmorphic facies, and intellectual disability (ID) that was associated with elevated lactate and alanine levels. At 3.5 years, she was just learning to walk and talk, while at 5 years, she had persistent delayed development and neurological issues (hypotonia and broad‐based gait) [4]. Protein analysis of her fibroblasts showed increased MRPS14 protein expression, as well as deficiencies in several assembled OXPHOS complexes. Specific reductions in mitochondrial‐encoded proteins, including cytochrome c oxidase II (COX2, encoded by *MT‐*CO_2_) were also present [4]. In this report, we report a second individual with novel compound heterozygous variants in *MRPS14* (c.109G>A; p.Asp37Asn and c.178A>G; p.Asn60Asp). This subject presented at 2 years of age with motor and language delays along with elevated serum lactate and alanine levels. Neuroimaging was consistent with Leigh Syndrome (also known as subacute necrotizing encephalomyelopathy). Laboratory evaluation showed decreased MRPS14 and COX2 protein levels.

## 2 Materials and Methods

Please see Supporting Information S1.

## 3 Results

### 3.1 Patient

The proband was a 2‐year‐old male that presented with a history of motor and language delays. He was the first child of his parents, who were both 26 years old at the time of his birth. Their pregnancy history included one later miscarriage followed by the birth of an unaffected younger brother. His mother was of Vietnamese descent and his father was of Belgian/German descent. Family history included a paternal aunt with adolescent‐onset epilepsy.

**FIGURE 1:**
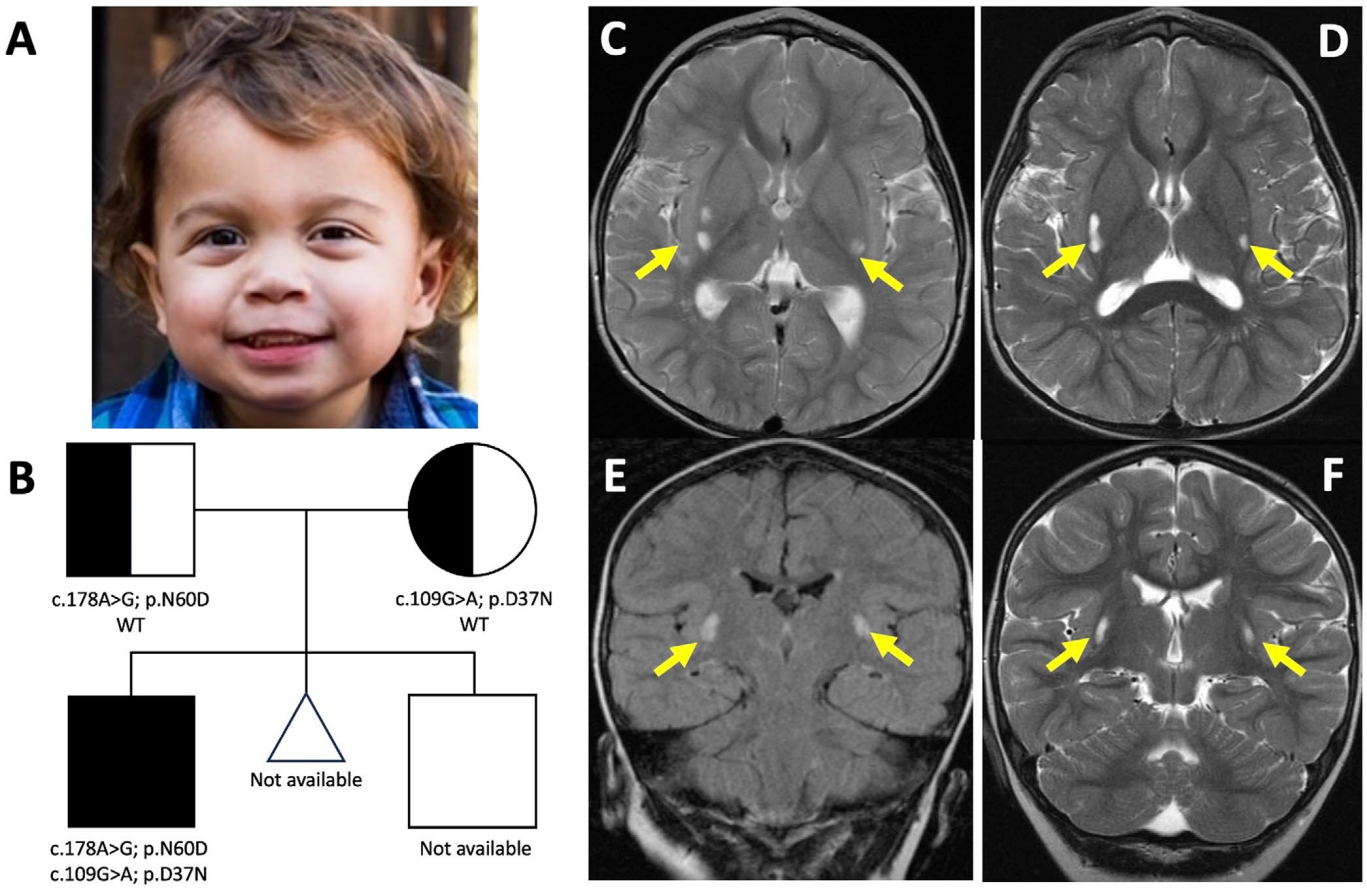
(A) Subject had facial features that included mild prominence of the glabella, a mildly down‐slanting palpebral fissures, and a mildly deep nasal bridge. (B) Pedigree showing maternal inheritance of the p.D37N variant and paternal inheritance of the p.N60D variant (genotype of sibling and miscarried child not available). (C–E) MRI brain at (C, E) ~2.5 years and (D, F) 5 months later. Axial T2‐weighted images (C, D) and coronal FLAIR (E) and T2‐weighted images (F) showed oval‐shaped hyperintense abnormalities situated along the posterior margin of both putamen and within the external capsule. There was a single focus on the left and two foci on the right (yellow arrows) that became more confluent and vacuolar with time. None of these lesions enhanced with contrast or were associated with diffusion restriction.

The pregnancy was complicated by in utero anemia of unknown etiology that required two blood transfusions. Labor was induced at 35 weeks, and growth parameters at birth had weight and length at the 4th‐ and 13th‐percentile (using Fenton preterm growth charts), respectively. He was admitted to the NICU for monitoring over a 2.5‐week period and did not require any additional transfusions/interventions. At 2 years, he was seen in our Pediatric Neurogenetics Clinic for developmental delays. At that time, he was at the 22nd‐, 5th‐, and 25th‐percentile for weight, height, and head circumference, respectively. He was alert and made good eye contact. He babbled but had no appreciable expressive or receptive language. He had normal muscle bulk with spasticity and increased tone that was more severe in his lower extremities. He could pull himself up to stand and cruised by toe‐walking. At 8 years, he was well‐developed and had facial features that included mild prominence of the glabella, down‐slanting palpebral fissures, mildly prominent upper central incisors (secondary), and a mildly deep nasal bridge (Figure 1A). He spoke with somewhat comprehensible simple sentences and walked with a walker.

### 3.2 Laboratory Testing and Neuroimaging

Laboratory evaluations at 2.5 years revealed elevated alanine (551; 157–481 mmol/L), lactate (31.4; 9.0–16.0 mmol/L), and an elevated lactate/pyruvate ratio (78; 10–20). Brain MRIs at 2.5 and 3 years were consistent with Leigh syndrome and showed progressive signal abnormalities along the posterior margin of the putamen and the external capsules (Figure 1C) that were more vacuolar and confluent on the right. An elevated lactate peak was seen with MRI spectroscopy (see Figure S1).

### 3.3 Exome Sequencing

**FIGURE 2:**
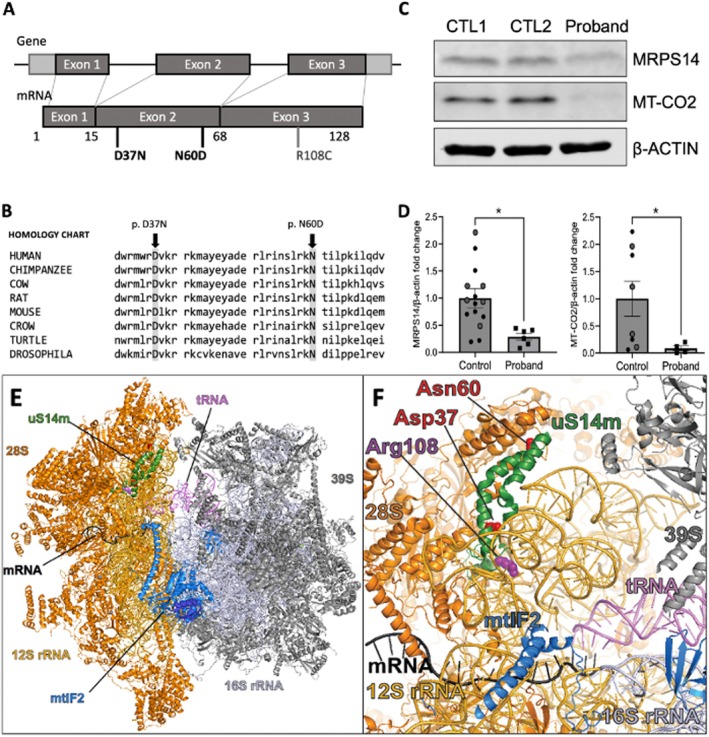
(A) MRPS14 gene and protein schematics and variant locations. (B) the p.Asp37Asn and p.Asn60Asp variants involved amino acids that were conserved across multiple vertebrate/invertebrate species. (C) representative image of Western blots, 60ug of cellular protein was loaded in each well and subsequently evaluated with primary antibodies for MRPS14 and MT‐CO_2_. Beta‐actin was used as a loading control and each blot normalized to controls. (D) Graphic representation of data from Western blots utilizing four independent experiments (two‐group comparison with non‐parametric Mann Whitney U test; *p < 0.05). (E, F) Modeling of uS14m (MRPS14) mutation locations p.Asp37Asn and p.Asn60Asp and comparison the previously published p.Arg108Cys location within the 55S mitochondrial initiating ribosome. Human mitochondrial initiating ribosome structure (PDB 6GAW) (E) The complete structure of the initiating 55S ribosome. The ribosomal proteins from the small subunit are shown and labeled in orange, and the 12S rRNA in yellow. The ribosomal proteins from the large subunit are shown in gray, and the 16S rRNA is shown in light gray. The mRNA is shown in black, and the tRNA in lavender. The mitochondrial initiation factor mtIF2 is shown in blue. Ribosomal protein uS14m is shown in green. In silico modeling used PDB model 6GAW and was visualized with PyMol Version 2.5.8 (Schrödinger LLC, New York, NY). The blue frame indicates the part of the structure that is magnified and shown in (F).

Exome sequencing of samples from the subject and both parents identified two novel bi‐allelic *MRPS14* compound heterozygous missense variants. The maternally (c.109G>A; p.Asp37Asn) and paternally (c.178A>G; p.Asn60Asp) inherited variants were both located within exon 2 (Figure 2A,B). No other pathogenic variants were identified. These Asp37Asn and Asn60Asp variants were present in gnomAD v4.1.1 on one and three alleles (of 239,348 and 250,220 total, respectively; see Supporting Information S1 for additional data regarding variants) (no homozygous individuals were present for either variant in gnomADv4.1). Furthermore, these variants had PhyloP100way scores of 7.598 and 7.814 and EIGEN pathogenicity predictions of 0.8682 (Pathogenic Moderate) and 0.6329 (Uncertain), respectively. In addition to the MRPS14 variants found in trans, a maternally inherited *PHF8* variant (c.2969 G>A; p.S990N) was also identified and classified as likely benign. Sequence analysis and deletion testing of the mitochondrial genome were performed separately with no genomic variants identified.

### 3.4 Protein Analysis

Western blots of fibroblast protein extracts revealed a significant decrease in the levels of both MRPS14 and COX2 proteins in our proband compared to control lines (Figure 2C,D). Protein modeling demonstrated localization of both variants in relative relation to the previously reported Arg108Cys pathogenic variant on the uS14 protein. Both variants are located in proximity to the tRNA, mRNA, and mitochondrial initiation factor‐2 within the ribosomal complex (mtIF2; Figure 2E,F).

## 4 Discussion

We report a subject with novel bi‐allelic *MRPS14* variants who presented at 2 years of age with significant motor/language delays, increased tone, and ID that was associated with elevated levels of serum lactate and alanine. Neuroimaging was consistent with Leigh syndrome and demonstrated progressive bilateral basal ganglia lesions. MR brain spectroscopy had increased lactate. A previous report noted Subject‐R108C possessed homozygous *MRPS14* variants (c.322C>T; p.R108C) that were associated with diffuse hypotonia, hypertrophic cardiomyopathy, cognitive disability, motor delays, along with elevated serum lactate and alanine levels (no neuroimaging results were reported) [4]. Her parents were distantly related heterozygous carriers of the variant [4], with both having issues that could be associated with mitochondrial dysfunction (rhabdomyolysis and spontaneous abortions) implying the variant could possess partial dominant‐negative effects. Because of the parent's mild consanguinity, it was unknown whether homozygous variants in other genes could have a role in their daughter's phenotype. The subject reported here has bi‐allelic variants that were inherited from his unrelated and seemingly unaffected parents. He also presented with similar clinical findings as Subject‐R108C, along with diffuse spasticity and in utero anemia (although cardiac function was within normal limits).

The features of mitochondrial disease can include a spectrum of early‐onset cardiac dysfunction, hypotonia, developmental delay, and structural brain abnormalities associated with increased lactate levels [11]. Leigh Syndrome can be quite heterogeneous and has a wide diagnostic criteria that includes the presence of neuroimaging or neuropathologic lesions combined with: (i) delay of intellectual and motor development, (ii) abnormal energy metabolism indicated by elevated lactate serum or cerebrospinal fluid, and (iii) clinical evidence of dysfunction of the brainstem and/or basal ganglia [12]. Neuroimaging abnormalities can include necrotizing lesions of the deep gray matter and metabolic strokes (also be seen with Wernicke encephalopathy due to thiamine deficiency as thiamine is a cofactor of pyruvate dehydrogenase) [7, 13]. Leigh syndrome is often associated with mitochondrial deficiencies that can be the result of over 100 different monogenetic disorders associated with mitochondrial OXPHOS dysfunction [12], although to date only two mitochondrial ribosomal subunit genes, *MRPL44* and *MRPS34*, have been associated with Leigh syndrome [7, 14].

*MRPS14* encodes a protein with a critical role in mitochondrial translation [4]. Previous MRPS14 modeling studies predicted its location to be in the mRNA channel of mt‐ribosomes [4]. This suggests that MRPS14 is in close contact with both the mt‐mRNA and the 12S rRNA, which may indicate its importance in guiding leaderless mt‐mRNAs into mt‐ribosomes (Figure 2E,F). In this context, amino‐acid substitutions in MRPS14 could have a dramatic effect on the efficiency of mt‐translation and alter mitochondrial output. Alternatively, these substitutions could decrease the stability of the mutant MRPS14 protein, leading to a deficiency in mt‐ribosomes along with a subsequent deficiency in mt‐translation. Protein analysis of Subject‐R108C's cells showed elevated expression of mutant MRPS14‐R108C, which was associated with deficiencies in assembled OXPHOS complexes. The most significant decreases were found in complexes I and IV (C1 & CIV), with protein levels of COX1 (*MT‐CO1*) and COX2 (*MT‐CO_2_*) also being significantly decreased [4]. We found that COX2 was also decreased in the subject reported here; however, in contrast to Subject‐R108C, this subject's MRPS14 levels were significantly decreased. This suggests that one or both of this subject's variants produced an unstable protein, while Subject‐R108C's upregulated MRPS14 protein was likely a more stable version of a dysfunctional protein.

Clinical care of this *MRPS14*‐related disorder (MRPS14‐RD) should consist of continued cardiac, ophthalmological, neurological, and neuroimaging evaluations. Although neither of these reported subjects has had any developmental regressions, surveillance should be particularly vigilant with any acute illness to monitor for that potential. Further subjects will likely provide more data regarding the phenotypes and required care needed for individuals with pathologic *MRPS14* variants. Interestingly, very few variants have been reported in the different mt‐ribosomal subunits, indicating these mt‐ribosomal proteins may be essential for development and deficient activity may result in early embryonic death [4]. Identifying more patients may be difficult, but it is critical to determining consistent and inconsistent phenotypic patterns associated with MRPS14‐related disease.

## Author Contributions

M.G.O., C.F., Z.M., M.C., and T.M.P. designed the experiments and wrote the manuscript. M.G.O., Z.M., M.C., E.C., R.S., and M.B. performed the biological experiments and analyzed biological data. C.F., S.Y.Z., R.G., M.D., F.M., and T.M.P. collected clinical information and evaluated the patients, provided clinical assessments, and whole‐exome sequencing data. All authors discussed the results and implications and commented on the manuscript.

## Conflicts of Interest

The authors declare no conflicts of interest.

## Supporting information


Data S1.

## Data Availability

The data that support the findings of this study are available from the corresponding author upon reasonable request.

## References

[1] P. Smits , J. Smeitink , and L. van den Heuvel , “Mitochondrial Translation and Beyond: Processes Implicated in Combined Oxidative Phosphorylation Deficiencies,” Journal of Biomedicine & Biotechnology 2010 (2010): 737385. 20396601 10.1155/2010/737385 PMC2854570

[2] A. V. Kotrys and R. J. Szczesny , “Mitochondrial Gene Expression and Beyond‐Novel Aspects of Cellular Physiology,” Cells 9, no. 1 (2019). 10.3390/cells9010017 PMC7017415 31861673

[3] P. Fernández‐Silva , J. A. Enriquez , and J. Montoya , “Replication and Transcription of Mammalian Mitochondrial DNA,” Experimental Physiology 88, no. 1 (2003): 41–56. 12525854 10.1113/eph8802514

[4] C. B. Jackson , M. Huemer , R. Bolognini , et al., “A Variant in MRPS14 (uS14m) Causes Perinatal Hypertrophic Cardiomyopathy With Neonatal Lactic Acidosis, Growth Retardation, Dysmorphic Features and Neurological Involvement,” Human Molecular Genetics 28, no. 4 (2019): 639–649. 30358850 10.1093/hmg/ddy374

[5] S. Aibara , V. Singh , A. Modelska , and A. Amunts , “Structural Basis of Mitochondrial Translation,” eLife 9 (2020). 10.7554/eLife.58362 PMC7438116 32812867

[6] B. J. Greber , D. Boehringer , M. Leibundgut , et al., “The Complete Structure of the Large Subunit of the Mammalian Mitochondrial Ribosome,” Nature 515, no. 7526 (2014): 283–286. 25271403 10.1038/nature13895

[7] N. J. Lake , B. D. Webb , D. A. Stroud , et al., “Biallelic Mutations in MRPS34 Lead to Instability of the Small Mitoribosomal Subunit and Leigh Syndrome,” American Journal of Human Genetics 101, no. 2 (2017): 239–254. 28777931 10.1016/j.ajhg.2017.07.005 PMC5544391

[8] A. R. D'Souza and M. Minczuk , “Mitochondrial Transcription and Translation: Overview,” Essays in Biochemistry 62, no. 3 (2018): 309–320. 30030363 10.1042/EBC20170102 PMC6056719

[9] S. E. Calvo , K. R. Clauser , and V. K. Mootha , “MitoCarta2.0: An Updated Inventory of Mammalian Mitochondrial Proteins,” Nucleic Acids Research 44, no. D1 (2016): D1251–D1257. 26450961 10.1093/nar/gkv1003 PMC4702768

[10] M. Schubert Baldo and L. Vilarinho , “Molecular Basis of Leigh Syndrome: A Current Look,” Orphanet Journal of Rare Diseases 15, no. 1 (2020): 31. 31996241 10.1186/s13023-020-1297-9 PMC6990539

[11] T. Gardeitchik , M. Mohamed , B. Ruzzenente , et al., “Bi‐Allelic Mutations in the Mitochondrial Ribosomal Protein MRPS2 Cause Sensorineural Hearing Loss, Hypoglycemia, and Multiple OXPHOS Complex Deficiencies,” American Journal of Human Genetics 102, no. 4 (2018): 685–695. 29576219 10.1016/j.ajhg.2018.02.012 PMC5985281

[12] N. J. Lake , A. G. Compton , S. Rahman , and D. R. Thorburn , “Leigh Syndrome: One Disorder, More Than 75 Monogenic Causes,” Annals of Neurology 79, no. 2 (2016): 190–203. 26506407 10.1002/ana.24551

[13] N. Latt and G. Dore , “Thiamine in the Treatment of Wernicke Encephalopathy in Patients With Alcohol Use Disorders,” Internal Medicine Journal 44, no. 9 (2014): 911–915, 10.1111/imj.12522. 25201422

[14] F. Distelmaier , T. B. Haack , C. B. Catarino , et al., “MRPL44 Mutations Cause a Slowly Progressive Multisystem Disease With Childhood‐Onset Hypertrophic Cardiomyopathy,” Neurogenetics 16, no. 4 (2015): 319–323. 25797485 10.1007/s10048-015-0444-2

