# Supplementary material for: Compound Heterozygous MRPS14 Variants Associated With Leigh Syndrome
Source: Ann Clin Transl Neurol. 2025 May 2;12(8):1693–7. doi: 10.1002/acn3.70065 (PMC12343306; doi:10.1002/acn3.70065)
Supplement: Supplementary file 1 — Data S1. [file ACN3-12-1693-s001.docx]

**SUPPLEMENTARY MATERIAL**

**PATIENTS AND METHODS**

**Informed consent and genetic evaluation.**

Standard protocol approvals, registrations, and patient consents. Research protocols were approved through the institutional review board, and the family gave informed consent (CSMC IRB protocol Pro00037131).

**Skin biopsy and fibroblast culture**

Fibroblasts were generated from patient skin biopsies and were grown at 37°C under 5% CO2 in Dulbecco’s Modified Eagles Medium (Thermo Fisher Scientific, Waltham, MA) supplemented with 10% bovine serum and 1% of penicillin/streptomycin (Thermo Fischer Scientific).

**Exome and Mitochondrial sequencing**

Using genomic DNA from the proband and parents, the exonic regions and flanking splice junctions of the genome were captured using the IDT xGen Exome Research Panel v1.0 (Integrated DNA Technologies, Coralville, IA). Massively parallel (NextGen) sequencing was done on an Illumina system with 100bp or greater paired end reads. Reads were aligned to human genome build GRCh37/UCSC hg19 and analyzed for sequence variants using a custom-developed analysis tool. Reported variants were confirmed, if necessary, by an appropriate orthogonal method in the proband and, if submitted, in selected relatives. Additional sequencing technology and variant interpretation protocol has been previously described.^1^ The general assertion criteria for variant classification are publicly available on the GeneDx ClinVar submission page (http://www.ncbi.nlm.nih.gov/clinvar/submitters/26957/)"

For mitochondrial genome sequencing and deletion analysis, the entire mitochondrial genome was amplified by long-range PCR and sequenced using next-generation sequencing as previously described.^2^ DNA sequence was aligned to the revised Cambridge Reference Sequence (rCRS, GeneBank sequence NC_012920) and compared with the reported variants and polymorphisms listed in the MITOMAP database (http://www.mitomap.org). Reportable variants including pathogenic variants, likely pathogenic variants and variants of uncertain significance were classified according to a modified version of Specifications of the ACMG/AMP standards and guidelines for mitochondrial DNA variant interpretation.^3^

**Western blots**

Fibroblasts from two control (CTL) and our MRPS14 subjects (MRPS14) were expanded in Dulbecco’s Modified Eagles Medium (Thermo Fisher Scientific, Waltham, MA) supplemented with 10% bovine serum and 1% of penicillin/streptomycin (Thermo Fischer Scientific, Waltham, MA). Cells were then incubated with TrypLE (Thermo Fischer Scientific, Waltham, MA) for 5 minutes at 37 °C washed with PBS and centrifuged at 1000 RPM for 3 minutes. Pellets were frozen at −80 °C. Thawed samples were then lysed using 1X NETN buffer (20 mM Tris-HCl (pH 8.0), 100 mM NaCl, 0.5 mM EDTA and 0.5% NP-40) supplemented with phosphatase–protease inhibitor cocktail (MS-SAFE, Sigma-Aldrich). Lysates were sonicated in an automated cold bath sonicator for 20 min using alternating 10 second pulses followed by 10 second rests. Samples were centrifuged for 20 min at 4 °C at 15,000 RPM. Total soluble protein concentrations were measured using BCA (Thermo Fischer Scientific, Waltham, MA). Then, 4X Laemmli sample buffer (161-0774; Bio-Rad, Hercules, CA) was added to 60 μg of total protein extracts and samples were boiled for 10 min. Samples were run in 4–20% Mini-PROTEAN TGX precast gels (456–1094; Bio-Rad, Bio-Rad, Hercules, CA) and transferred to polyvinylidene difluoride (PVDF) membranes using the Trans-Blot Turbo Transfer System (Bio-Rad, Bio-Rad, Hercules, CA). Membranes were blocked with Odyssey blocking buffer (LI-COR, Lincoln, NE) and then incubated with primary antibodies overnight at 4 °C. Following incubation with dye-labeled secondary antibodies for 2 h at room temperature, signals were visualized using an Odyssey Fc imaging system (LI-COR, Lincoln, NE). Primary antibodies used were human anti-MRPS14 (HPA051087; Sigma-Aldrich, St. Louis MO) and anti-MT-CO2 (ab110258; Abcam, Waltham, MA) and β-Actin (A5441, Sigma-Aldrich, Saint Louis MO). Secondary antibodies were IRDye 680 RD goat anti-mouse and IRDye 800CW goat anti-rabbit (926-68070 and 926-32211, respectively; LI-COR, Lincoln, NE) at a dilution of 1:5,000. Plots were calculated by first normalizing bands to β-Actin and then to the CTL signal present in each blot using ImageJ.

**Statistical analysis**

Statistical analysis was performed using non-parametric Mann Whitney U test for two-group comparison using Prism9 (GraphPad Software, San Diego, CA), with p<0.05 considered significant. Values were expressed as mean ± SEM.

**In Silico Modeling**

In silico modelling used PDB model 6GAW and was visualized with PyMol Version 2.5.8 (Schrödinger, LLC, New York, NY).^4^

**RESULTS**

**
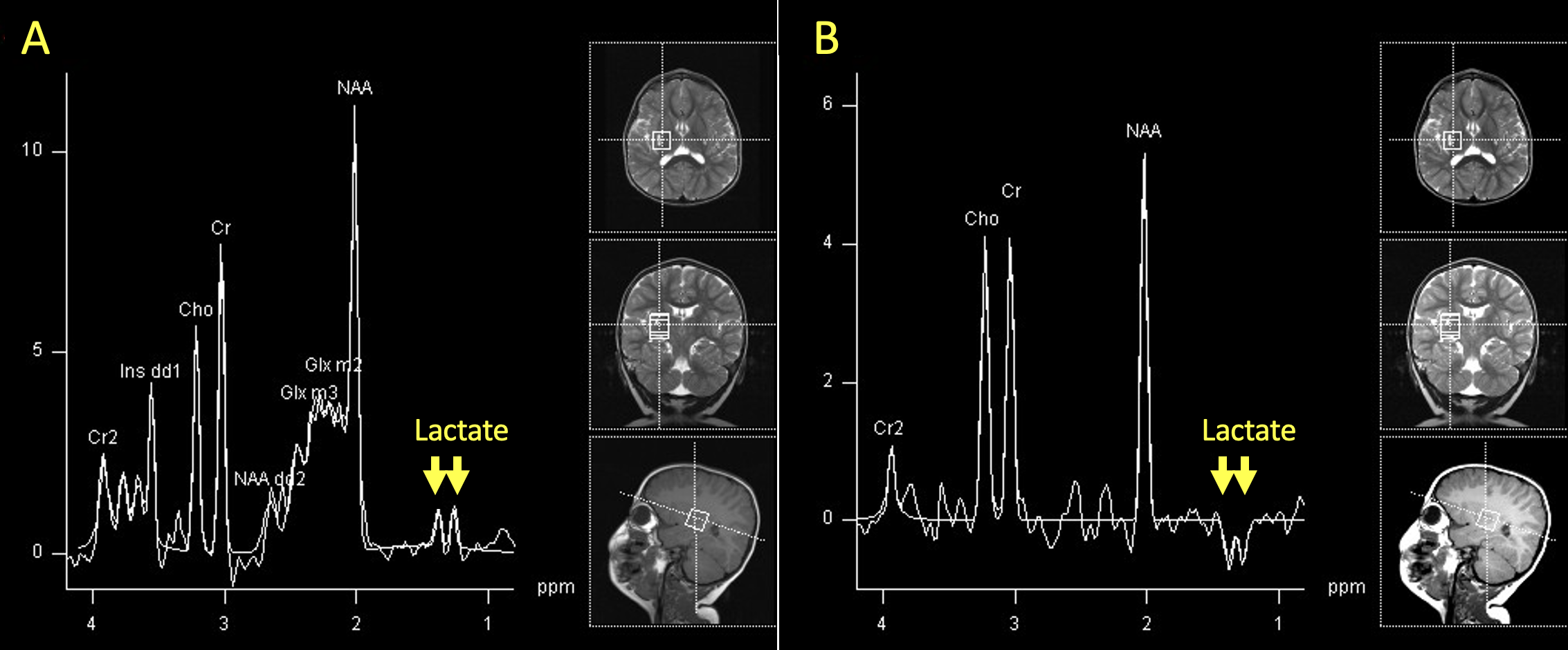
**

**Supplementary Figure 1.** **Short (A) and long (B) echo single voxel MR Spectroscopy with the voxel placed on the abnormal appearing Basal Ganglion. The abnormal lactate doublet peaks demonstrate the classic inversion of the signal on the long echo acquisition.**

**SUPPLEMENTARY MATERIAL REFERENCES**

^1^Retterer K, Juusola J, Cho MT, Vitazka P, Millan F, Gibellini F, Vertino-Bell A, Smaoui N, Neidich J, Monaghan KG, McKnight D, Bai R, Suchy S, Friedman B, Tahiliani J, Pineda-Alvarez D, Richard G, Brandt T, Haverfield E, Chung WK, Bale S. [Clinical application of whole-exome sequencing across clinical indications.](https://pubmed.ncbi.nlm.nih.gov/26633542/) Genet Med. 2016 18:696-704. doi: 10.1038/gim.2015.148. Epub 2015 Dec 3.PMID: 26633542

^2^Bai R, Cui H, Devaney JM, Allis KM, Balog AM, Liu X, Schnur RE, Shapiro FL, Brautbar A, Estrada-Veras JI, Hochstetler L, McConkie-Rosell A, McDonald MT, Solomon BD, Hofherr S, Richard G, Suchy SF. I[nterference of nuclear mitochondrial DNA segments in mitochondrial DNA testing resembles biparental transmission of mitochondrial DNA in humans.](https://pubmed.ncbi.nlm.nih.gov/33846581/) Genet Med. 2021 Aug;23(8):1514-1521. doi: 10.1038/s41436-021-01166-1. Epub 2021 Apr 12.PMID: 33846581

^3^McCormick EM, Lott MT, Dulik MC, Shen L, Attimonelli M, Vitale O, Karaa A, Bai R, Pineda-Alvarez DE, Singh LN, Stanley CM, Wong S, Bhardwaj A, Merkurjev D, Mao R, Sondheimer N, Zhang S, Procaccio V, Wallace DC, Gai X, Falk MJ. [Specifications of the ACMG/AMP standards and guidelines for mitochondrial DNA variant interpretation.](https://pubmed.ncbi.nlm.nih.gov/32906214/) Hum Mutat. 2020 41:2028-2057. doi: 10.1002/humu.24107. Epub 2020 Nov 10.PMID: 32906214

^4^Kummer E, Leibundgut M, Rackham O, Lee RG, Boehringer D, Filipovska A, Ban N. (2018) Unique features of mammalian mitochondrial translation initiation revealed by cryo-EM. Nature. 560:263-267. doi: 10.1038/s41586-018-0373-y. PMID: 30089917
